# Lack of Cell Cycle Inhibitor p21 and Low CD4^+^ T Cell Suppression in Newborns After Exposure to IFN-β

**DOI:** 10.3389/fimmu.2021.652965

**Published:** 2021-04-12

**Authors:** Jop Jans, Wendy W. Unger, Elisabeth A. M. Raeven, Elles R. Simonetti, Marc J. Eleveld, Ronald de Groot, Marien I. de Jonge, Gerben Ferwerda

**Affiliations:** ^1^ Laboratory of Pediatrics, Division of Pediatric Infectious Diseases and Immunology, Erasmus MC University Medical Center-Sophia Children’s Hospital, Rotterdam, Netherlands; ^2^ Laboratory of Medical Immunology, Section Pediatric Infectious Diseases, Department of Laboratory Medicine, Radboud Institute for Molecular Life Sciences, Radboud University Medical Center, Nijmegen, Netherlands; ^3^ Radboud Center for Infectious Diseases, Radboud University Medical Center, Nijmegen, Netherlands

**Keywords:** interferon beta, respiratory syncytial virus, newborns, immunity, CD4 T cells, proliferation, cell cycle, cyclic-dependent kinase inhibitor/p21

## Abstract

Type I IFNs, such as interferon alpha and interferon beta, are key regulators of the adaptive immune response during infectious diseases. Type I IFNs are induced upon infection, bind interferon α/β receptors on T-cells and activate intracellular pathways. The activating and inhibitory consequences of type I IFN-signaling are determined by cell type and cellular environment. The neonatal immune system is associated with increased vulnerability to infectious diseases which could partly be explained by an immature CD4^+^ T-cell compartment. Here, we show low IFN-β-mediated inhibition of CD4^+^ T-cell proliferation, phosphorylation of retinoblastoma protein and cytokine production in human newborns compared to adults. In addition, both naïve and total newborn CD4^+^ T-cells are unable to induce the cell-cycle inhibitor p21 upon exposure to IFN-β in contrast to adults. The distinct IFN-β-signaling in newborns provides novel insights into T cell functionality and regulation of T cell-dependent inflammation during early life immune responses.

## Introduction

Newborns encounter a large variety of infectious microorganisms during the first months of life. Whereas antibodies are transferred from the mother leading to passive protection, T cell-mediated immunity has to be developed by the newborn itself. A delicate balance between accurate T cell activation and prevention of T cell-mediated immunopathology is essential to mount a protective immune response. Clinical presentation and disease progression of many infectious diseases are different in newborns compared to adults, which raises the question which regulatory mechanisms of T-cell-mediated inflammation are present in newborns. The low production of cytokines such as interferon gamma (IFN-γ) in newborns indicates a reduced activation of CD4^+^ T cells (1). On the other hand, activated CD4^+^ T cells and T cell-mediated immunopathology has been observed during neonatal sepsis (2) and severe viral infections, including neonatal RSV infections (3). Type I IFNs, such as interferon alpha (IFN-α) and interferon beta (IFN-β), are key regulators of the adaptive immune response, including CD4^+^ T cells (4). Type I IFNs bind the interferon α/β receptor (IFNAR1/2) and induce a variety of intracellular signals. Type I IFNs can directly inhibit or activate CD4^+^ T cells and thereby affect the proliferation and cytokine production by CD4^+^ T cells (4). For instance, type I IFNs inhibit proliferation of T cells during viral infections in mice (5). In addition, IFN-β inhibits the proliferation of adult T cells *in vitro (* 6) and treatment of adults with multiple sclerosis with IFN-β inhibits the ex vivo proliferation of CD4^+^ T cells (7). However, no study so far compared the effects of IFN-β on newborns with adults. We hypothesize that the regulation of CD4^+^ T cells by type I IFNs is different in newborns compared to adults. The goal of this study was to determine the differential effects of IFN-β on newborn CD4^+^ T cell functionality compared to adults. Newborn and adult mononuclear cells (MCs) were used to characterize IFN-β-dependent and RSV-induced regulation of T cells. Isolated CD4^+^ T cells, including naïve CD4^+^ T cells, were collected to gain insights into T cell-intrinsic mechanisms in newborns and adults that depend on type I IFNs. Dissecting the effects of type I IFNs and IFNAR signaling in newborn and adult CD4^+^ T cells will provide novel insights into the ontogeny of T cell functionality and regulation of T-cell-mediated inflammation during early life immune responses.

## Materials and Methods

### Virus Culture

Green fluorescent protein (GFP)-labeled RSV A2 (rgRSV30), kindly proved by Dr. M.E. Peeples, was cultured on HeLa cells (ATCC, CCL-2) as previously described (8). The virus stock was snap-frozen and stored at -80°C.

### Cell Isolation

After cesarean section delivery of the placenta, human newborn cord blood was collected from the umbilical cord. Premature births and birth to HIV-positive mothers were excluded. Peripheral blood was collected form healthy adult volunteers. Human experimental guidelines of the Regional Committee on Research involving Human Subjects Arnhem-Nijmegen, were observed, following protocols approved by the local institutional review boards. Newborn or adult blood was layered onto Lymphoprep (Stemcell Technologies) to collect CBMCs and PBMCs, respectively. CD4^+^ and CD8^+^ T cells were isolated from MC fractions by positive selection with magnetic CD4 and CD8 microbeads, respectively, according to the manufacturer’s instructions (Miltenyi Biotec, Auburn, CA). Purity of CD4^+^ or CD8^+^ T cell preparations was consistently > 93%. When indicated, CD25^+^ cells were depleted from isolated T cells. For this, untouched CD4^+^ T cells were isolated from whole blood (RosetteSep CD4 Enrichment Kit; Stemcell Technologies) and subsequently CD25^high^ T cells were depleted using magnetic CD25 microbeads (Miltenyi Biotec, Auburn, CA). More than 80% of CD25^+^ T cells were depleted as confirmed by flow cytometry (**Supplementary Figure 2C**). Naïve CD4^+^ T cells were isolated from MC fraction by negative selection (Naïve CD4^+^ T cell isolation kit II, human; Miltenyi Biotec, Auburn, CA) followed by a positive selection with magnetic CD27^+^ microbeads to remove terminally differentiated CD4^+^ T cells (9). RPMI 1640 medium (Invitrogen) + 10% fetal calf serum (FCS) + 2 mM L-Glutamine was used as culture medium.

### Cell Stimulation

Each 96-well plate was filled with 5 x 10^5^ CMBCs or PBMCs per well or with 1 x 10^5^ isolated newborn or adult T cells per well. In separate experiments, the supernatants from RSV-stimulated MCs (sup RSV-MC) were harvested, centrifuged and used for subsequent stimulation of cells from different donors. No viral particles, determined by fluorescent staining of RSV fusion protein, were detected after exposure of HeLa cells to supernatant from RSV-stimulated cells (data not shown). For the proliferation assay, MCs or isolated T cells were labeled with CFSE, respectively 5 µg/ml or 1 µg/ml. Cells were exposed to RSV (MOI 0.04, 0.2 or 1), recombinant human IFN-α (PBL Interferon Source, NJ) or recombinant human IFN-β (PBL Interferon Source, NJ) for 24h. Subsequently, MCs or isolated T cells were activated with respectively PHA (1 µg/ml; Sigma) or CD3/CD28 beads (Dynabeads ThermoFisher Scientific) for 72h. When indicated, the human interferon alpha/beta receptor (IFNAR2) was blocked with monoclonal antibodies against IFNAR2 (5 µg/ml, IgG2a, clone MMHAR-2, PBL Interferon Source, NJ) or isotype controls for 1h before stimulation. All conditions were performed in duplicate.

### Flow Cytometry

**Figure 1:**
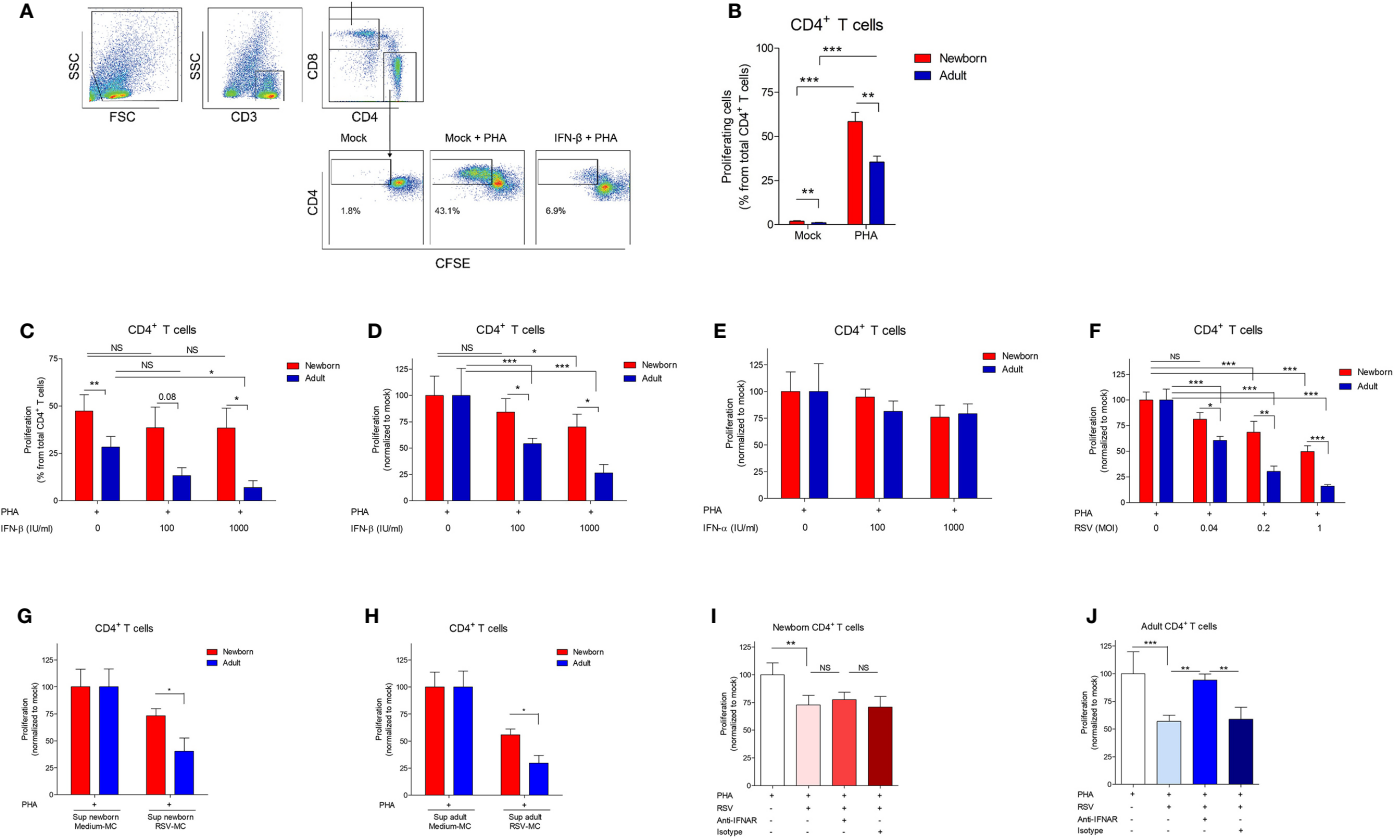
IFN-β-mediated inhibition of CD4 ^+^T cell proliferation is reduced in newborns. **(A)** Representative gating strategy: CD3^+^ cells were identified from MCs followed by CD4^+^ and CD8^+^ cells to identify respectively CD4^+^ T cells and CD8^+^ T cells. CFSE fluorescence was used to determine the percentage of proliferating T cells. **(B)** Percentage of proliferating CD4^+^ T cells after incubation of CBMC (newborn) or PBMC (adults) with PHA for 72h. (N=14-19). **(C–F)** CMBCs or PBMCs were pre-incubated with medium, IFN-β (C-D), IFN-α **(E)**, or RSV **(F)** for 24h and, subsequently, incubated with PHA for 72h to induce proliferation. Proliferation of CD4^+^ T cells after pre-incubation with medium was normalized for **(D–F)**. For this, the proliferation of T cells in the condition with only medium was set to 100%. (N=5-6). **(G, H)** CBMCs **(G)** or PBMCs **(H)** were pre-incubated with supernatant derived from MCs exposed to medium or supernatant derived from MCs exposed to RSV and, subsequently, incubated with PHA for 72h to induce proliferation. Proliferation of CD4^+^ T cells after pre-incubation with supernatant derived from MCs exposed to medium was set to 100%. (N=5-7). **(I, J)** CBMCs **(I)** or PBMCs **(J)** were incubated with medium, blocking antibodies against IFNAR2 or isotype controls for 1h followed by exposure to medium or RSV for 24h and PHA for 72h to induce proliferation. Proliferation of CD4^+^ T cells after pre-incubation with medium was set to 100%. (N=5-6). Data are presented as means ± SEM. NS = not significant. * = *P*<0.05. ** = *P*<0.01. *** = *P*<0.001. ns, not significant.

Proliferation was determined by CFSE fluorescence (**Figure 1A**). The following fluorochrome-labeled monoclonal antibodies were used for immunophenotyping: CD3 Alexa Fluor 647, CD4 PerCp-Cy5.5, CD8 APC-H7, CD45RA Pe-Cy7, CD27 V500, CD25 Alexa Fluor 700, CD127 V450 (BD Biosciences). Mean fluorescence intensity of IFNAR1 PE and IFNAR2 PE (PBL Interferon Source, NJ) were determined for the expression of IFNAR1 and IFNAR2. 7-Aminoactinomycin D (7-AAD) was used to measure cell death. Cells were analyzed with LSR II flow cytometer.

### Phosflow

Isolated CD4^+^ T cells were incubated with medium for 2h at 37°C to reduce background phosphorylation after which IFN-β was added. CytoFix (BD Biosciences) was added to the cells for 10 min at 37°C. Cells were washed with PBS/1%BSA and permeabilized with Perm Buffer III (BD Biosciences) for 30 min on ice. Cells were washed and labeled with the following monoclonal antibodies: STAT1(pY701)-PE and Rb(pS780)-Alexa Fluor 647; BD Biosciences) for 30 min at RT. Cells were analyzed with LSR II flow cytometer.

### Quantitative Real-Time Polymerase Chain Reaction (qRT-PCR)

After stimulation, cells were stored in lysis buffer containing 1% β-mercaptoethanol at -80 °C. RNA was extracted according to the manufacturer’s protocol (Macherey-Nagel). cDNA was synthesized by iScript (BioRad) and stored at -20°C. qRT-PCR was conducted on a CFX96 (BioRad) using Taqman gene expression assays (GAPDH: Hs02758991_g1, CDKN1A/p21: Hs00355782_m1, CDKN1B/p27: Hs01597599_m1, p53: Hs01034249_m1, IRF9 (Hs00196051_m1), STAT1: Hs01013996_m1, STAT2: Hs01013123_m1). The PCR program consisted of 5 min at 96°C, followed by 50 cycles of 15 s at 96°C and 45 s at 60°C. CFX Manager 3.0 (BioRad) was used for analysis. Data were normalized and ΔC_t_ was calculated by subtracting the C_t_ value of the housekeeping gene GAPDH from the C_t_ value of the target gene. Relative expression was calculated as followed: 2^-(ΔC_t)._ All conditions were measured in duplicate.

### Enzyme-Linked Immunosorbent Assay (ELISA)

Tumor necrosis factor (TNF; R&D Systems) and interleuking 10 (IL-10; Sanquin PeliKine) concentrations were measured in the cell supernatants by ELISA according to the manufacturer’s instructions with a lower limit of detection of 39 pg/ml. All conditions were measured in duplicate.

### Statistical Analysis

Statistical analyses employed the Wilcoxon matched-pairs signed rank test for paired analysis between two conditions and repeated measures ANOVA with Bonferroni’s Multiple Comparison Test for paired analysis between more than two conditions. Comparison between newborns and adults employed Mann–Whitney U test. Comparison between more than two groups employed the Kruskal–Wallis test followed by Dunn’s Multiple Comparison Test. Tests were considered significant if *P*<0.05. All statistical analyses were done with GraphPad Prism.

## Results

### Low IFN-β-Mediated Inhibition of T Cell Proliferation in Newborns

Cord blood mononuclear cells (CBMCs) and adult peripheral blood mononuclear cells (PBMCs) were used to assess whether human newborn T cells are differently modulated by type I IFNs compared to adults. The effect of type I IFNs on T cell proliferation was determined by exposing newborn and adult MCs to IFN-β or IFN-α prior to the induction of proliferation by phytohaemagglutinin (PHA) (**Figure 1A**). PHA-induced proliferation of CD4^+^ T cells was higher in newborns compared to adults (**Figure 1B**). In the absence of PHA, a small percentage of CD4^+^ T cells proliferated spontaneously, which was significantly higher in newborns (1.9% ± 0.3) compared to adults (1.1% ± 0.3) (**Figure 1B**). Exposure to IFN-β inhibited PHA-induced proliferation of adult CD4^+^ T cells in a dose-dependent fashion and the inhibitory effect of IFN-β was lower on newborn CD4^+^ T cells compared to adult CD4^+^ T cells (**Figure 1C**). Due to the high variance in PHA-induced proliferation between individuals, we normalized the data and observed similar results: the inhibitory effect of IFN-β was lower on newborn CD4^+^ T cells compared to adult CD4^+^ T cells (**Figure 1D**). Contrary, exposure to IFN-α had no effect on newborn and adult CD4^+^ T cell proliferation (**Figure 1E**). Comparable results were obtained for newborn and adult CD8^+^ T cells (**Supplementary Figures 1A–D**) indicating a low IFN-β-mediated inhibition of CD4^+^ and CD8^+^ T cell proliferation in newborns.

### Low RSV-Induced Inhibition of T Cells Is IFNAR-Dependent in Adults and in Newborns

Our group previously showed that exposure to RSV induces type I IFNs by newborn and adult MCs (10). To confirm the inhibitory role of type I IFNs during a clinically relevant viral infection in early life, the effect of RSV on T cell proliferation was investigated. Exposure to RSV induced a dose-dependent inhibition of CD4^+^ T cell proliferation in newborns and adults (**Figure 1F**). In line with our observation regarding IFN-β, the inhibition of CD4^+^ T cell proliferation after exposure to RSV was lower in newborns compared to adults (**Figure 1F**). Comparable results were obtained for newborn and adult CD8^+^ T cells (**Supplementary Figure 1E**). We, next, determined whether exposure to RSV inhibits T cell proliferation *via* soluble mediators and IFNAR signaling. MCs were exposed to RSV for 24h and the supernatant was stored. Subsequently, MCs from different donors were incubated with supernatant derived from RSV exposed MCs (RSV-MC) to investigate the effect of soluble mediators on T cell proliferation. Exposure to RSV-MC supernatant inhibits CD4^+^ T cell proliferation and the inhibitory effect was lower in newborn CD4^+^ T cells compared to adults (**Figures 1G, H**). In newborns, blocking of IFNAR2 did not affect the inhibitory effect of RSV on CD4^+^ T cell proliferation (**Figure 1I**). In adults, IFNAR2 signaling contributed to the inhibitory effect of RSV on CD4^+^ T cell proliferation (**Figure 1J**). Comparable results were obtained for CD8^+^ T cells (**Supplementary Figures 1F, G**). These data indicate that RSV-induced IFNAR2-dependent inhibition of T cells is only present in adults and not in newborns.

### Low IFN-β-Mediated Inhibition of Retinoblastoma Protein Phosphorylation and Proliferation in Isolated Newborn CD4^+^ T Cells

**Figure 2:**
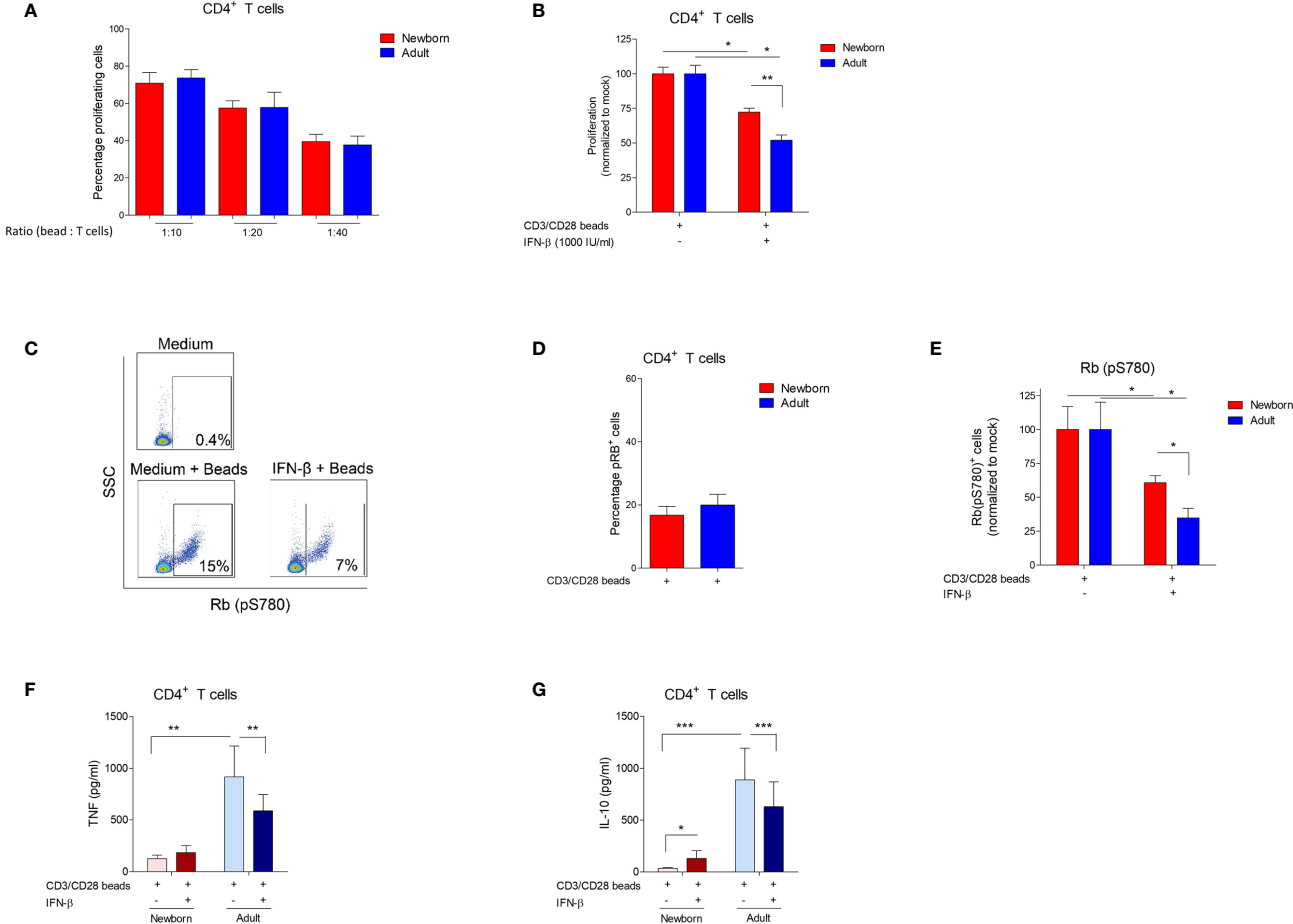
IFN-β-mediated inhibition of bead-induced CD4^+^ T cell proliferation, phosphorylation of Rb and cytokine production is reduced in newborns. **(A)** Percentage of proliferating CD4^+^ T cells after incubation of isolated adult and newborn CD4^+^ T cells with CD3/CD28 beads for 72h. (N=6-8). **(B)** Newborn and adult CD4^+^ T cells were pre-incubated with medium, or IFN-β for 24h and, subsequently, incubated with CD3/CD28 beads for 72h to induce proliferation. Proliferation after pre-incubation with medium was set to 100%. (N=6-8). **(C)** Representative gating strategy: CD4^+^ T cells were isolated from PBMC or CBMC and phosphorylation of Rb was determined. **(D)** Percentage of CD4^+^ T cells positive for Rb phosphorylation after incubation of isolated adult and newborn CD4^+^ T cells with CD3/CD28 beads for 72h. **(E)** Newborn and adult CD4^+^ T cells were pre-incubated with medium, or IFN-β for 24h and, subsequently, incubated with CD3/CD28 beads for 72h to induce phosphorylation of Rb. Phosphorylation of Rb after pre-incubation with medium was set to 100%. **(F, G):** Newborn and adult CD4^+^ T cells were pre-incubated with medium, or IFN-β for 24h and, subsequently, incubated with CD3/CD28 beads for 72h to induce cytokine production of TNF **(F)** and IL-10 **(G)**. Data are presented as means ± SEM. * = *P*<0.05. ** = *P*<0.01. *** = *P*<0.001.

To investigate the direct effect of IFN-β on CD4^+^ T cells, CD4^+^ T cells were isolated from MCs. Isolated CD4^+^ T cells were exposed to IFN-β and, subsequently, CD3/CD28 beads were used to stimulate the T cell receptor (TCR) and induce CD4^+^ T cell proliferation. TCR-induced proliferation of CD4^+^ T cells was similar between newborns and adults (**Figure 2A**). Exposure to IFN-β inhibits CD4^+^ T cell proliferation and the IFN-β-mediated inhibition was lower in newborns compared to adults (**Figure 2B**). These results are in line with our previous results regarding IFN-β-mediated inhibition of CD4^+^ T cell proliferation in a model of MCs. No increase of cell death was observed after exposure of CD4^+^ T cells to IFN-β (**Supplementary Figure 2A**). To further dissect the IFN-β-mediated CD4^+^ T cell inhibition, phosphorylation of retinoblastoma protein (Rb), an important requisite for T cell proliferation (11), was measured (**Figure 2C**). TCR-induced phosphorylation of Rb was similar between newborn and adult CD4^+^ T cells (**Figure 2D**). Exposure to IFN-β inhibits TCR-induced phosphorylation of Rb in CD4^+^ T cells and this inhibition by IFN-β was lower in newborn CD4^+^ T cells compared to adults (**Figure 2E**), which corresponds with the observed differences in CD4^+^ T cell proliferation between newborns and adults.

### IFN-β-Mediated Inhibition of Cytokine Release Is Absent in Newborn CD4^+^ T Cells

To evaluate the effect of IFN-β on other effector functions than CD4^+^ T cell proliferation and phosphorylation of Rb, CD4^+^ T cells were exposed to IFN-β and the capacity to produce pro- and anti-inflammatory cytokines, respectively tumor necrosis factor (TNF) and interleukin 10 (IL-10), was determined. TCR-induced production of TNF and IL-10 by newborn CD4^+^ T cells was low compared to adults (**Supplementary Figures 2B, C**). Exposure to IFN-β inhibits the TCR-induced production of TNF and IL-10 by adult CD4^+^ T cells, whereas no inhibitory effect of IFN-β on cytokine production by newborn CD4^+^ T cells was observed (**Figures 2F, G**). IL-10 production by regulatory CD4^+^ T cells (Tregs) has been implicated in the inhibition of conventional CD4^+^ T cells (12). The inhibitory effect of IFN-β on IL-10 production by adult T cells suggests that IFN-β-mediated inhibition of T cells most likely does not depend on the production of IL-10 by Tregs. To confirm these results, we depleted Tregs from CD4^+^ T cells (**Supplementary Figure 2D**) and showed that depletion of Tregs did not affect IFN-β-mediated inhibition of adult CD4^+^ T cell proliferation (**Supplementary Figures 2D, E**). These experiments show that the inhibitory effect of IFN-β on cytokine production by CD4^+^ T cells, a different effector function than proliferation, is also low in newborns compared to adults.

### IFN-β Induces Comparable Levels of Phosphorylated STAT1 and Transcription Factors in Newborn and Adult CD4^+^ T Cells

**Figure 3:**
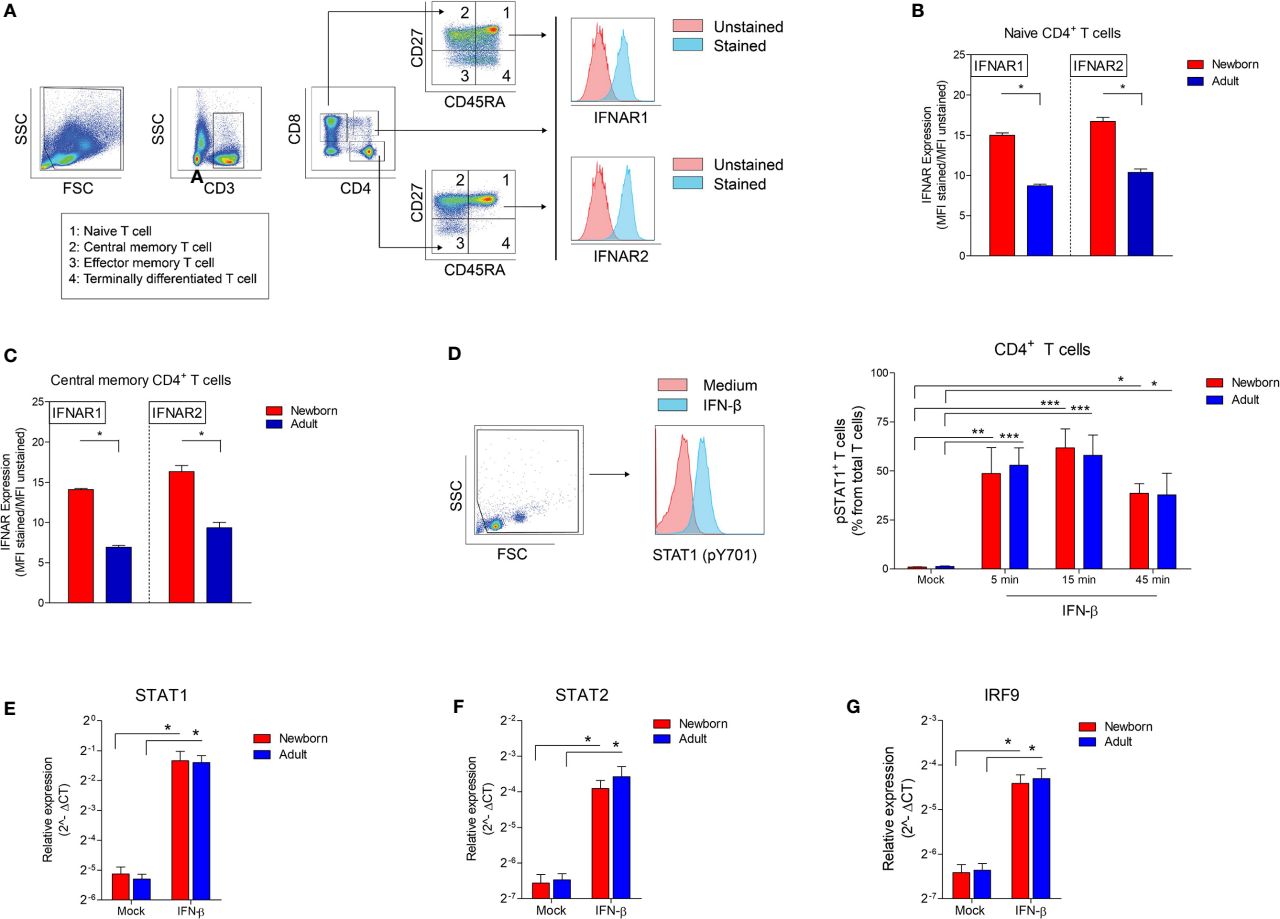
High IFNAR expression and similar induction of phosphorylated STAT1 and STAT1-induced transcription factors in newborn CD4^+^ T cells. **(A)** Representative gating to determine IFNAR1 and IFNAR2 expression on CD4^+^ T cell subsets. CD3, CD4 and CD8 was used to identify CD4^+^ T cells and CD8^+^ T cells. CD45RA and CD27 was used to identify T cell subsets: naïve (CD45RA^+^CD27^+^), central memory (CD45RA^-^CD27^+^), effector memory (CD45RA^-^CD27^-^) and terminally differentiated (CD45RA^+^CD27^-^) T cells. **(B)** Expression of IFNAR1 and IFNAR 2 on newborn and adults naïve **(B)** and central memory **(C)** T cells. (N=4-5). **(D)** Presence of phosphorylated STAT1 in newborn and adult CD4^+^ T cells upon exposure to IFN-β. (N=3-6). **(E–G)** Expression of **(E)** STAT1, **(F)** STAT2 and **(G)** IRF9 mRNA in newborn and adult CD4^+^ T cell after exposure to medium or IFN-β for 2h. (N=5-6). Data are presented as means ± SEM. * = *P*<0.05. ** = *P*<0.01. *** = *P*<0.001.

The observation of a low inhibitory effect on isolated newborn CD4^+^ T cells by IFN-β led to the hypothesis that IFN-β may differentially induce downstream signaling in newborn CD4^+^ T cells compared to adults. IFN-β binds IFNAR1 and IFNAR2, induces phosphorylation of signal transducer and activator of transcription 1 (STAT1) followed by transcription of multiple interferon-stimulated genes (ISG) (13). Therefore, we analyzed the expression of IFNAR on newborn and adult T cells and downstream signaling including phosphorylation of STAT1 and transcription of multiple ISG such as STAT1, STAT2 and interferon regulatory factor 9 (IRF9) mRNA (13). Using CD45RA and CD27 expression, we categorized CD4^+^ T cells into naïve (CD45RA^+^CD27^+^), central memory (CD45RA^-^CD27^+^), effector memory (CD45RA^-^CD27^-^) and terminally differentiated (CD45RA^+^CD27^-^) CD4^+^ T cells (**Figure 3A**) (9). The expression level of IFNAR1 and IFNAR2 was higher on newborn naïve CD4^+^ T cells and newborn central memory CD4^+^ T cells compared to adults (**Figures 3B, C**). IFNAR1/2 expression could not be determined on newborn effector memory and terminally differentiated T cells as these populations are barely present or undetectable in newborns (<0.01%) (**Supplementary Figures 3A, B**). In adults, IFNAR1 and IFNAR2 expression was higher on naïve T cells compared to effector memory and terminally differentiated T cells (**Supplementary Figures 3C, D**). STAT1 phosphorylation in CD4^+^ T cells was detected after 5 minutes exposure of isolated CD4^+^ T cells to IFN-β and comparable between newborn and adult CD4^+^ T cells (**Figure 3D**). STAT1 phosphorylation leads to nuclear accumulation and transcription of transcription factors STAT1, STAT2 and IRF9. Binding of these transcription factors to IFN-stimulated response element (ISRE) induces IFN type-I inducible genes (ISGs). Baseline expression and the induction of STAT1, STAT2 and IRF9 by IFN-β were comparable between newborn and adult CD4^+^ T cells (**Figures 3E–G**). These similarities between newborn and adult CD4^+^ T cells indicate that the expression of IFNAR and downstream signaling, such as the phosphorylation of STAT1 and the induction of transcription factors STAT1, STAT2 and IRF9 are not responsible for the observed low IFN-β-mediated T-cell inhibition in newborn CD4^+^ T cells.

### Contrary to Adults, Newborn Naïve CD4^+^ T Cells Lack the Induction of p21 After Exposure to IFN-β

**Figure 4:**
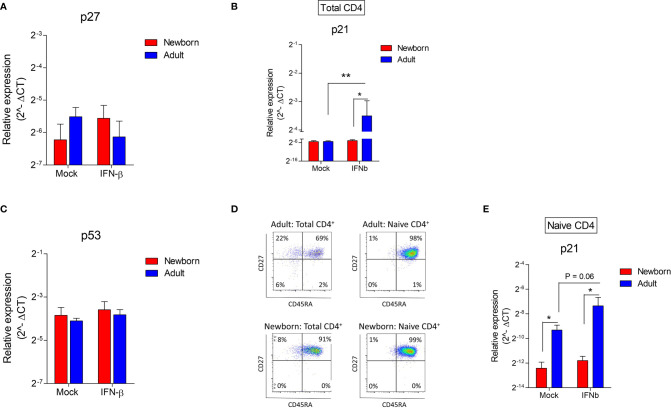
Contrary to adults, newborn naïve CD4^+^ T cells lack the induction of p21 after exposure to IFN-β. **(A–C)** Expression of p27, p21 and p35 after exposure of newborn and adult CD4^+^ T cells to IFN-β for 24h. (N=4-8). **(D)** Subset analysis after isolation of total and naïve CD4^+^ T cells in newborns and adults. **(E)** Expression of p21 after exposure of newborn and adult naïve CD4^+^ T cells. (N=4). Data are presented as means ± SEM. * = *P*<0.05. ** = *P*<0.01.

To elucidate our phenotype in which IFN-β exerts a low anti-proliferative effect on neonatal CD4^+^ T cells, the two most important IFN-induced cell cycle inhibitors that act as a major effector molecule of cell cycle arrest; cyclic-dependent kinase inhibitor (CDKI) p27 and p21 (14, 15) were investigated. IFN-β did not induce p27 mRNA in newborn and adult CD4^+^ T cells (**Figure 4A**). Newborn CD4^+^ T cells also lack the induction of p21, whereas p21 was induced in adult CD4^+^ T cells after exposure to IFN-β (**Figure 4B**). The expression of p21 is tightly regulated *via* p53-dependent and p53-independent pathways (14–16). IFN-β did not induce p53 mRNA in newborn and adult CD4^+^ T cells (**Figure 4C**), indicating that in our assay the transcription of p53 is not affected by exposure of newborn or adult CD4^+^ T cells to IFN-β. A major difference between neonatal and adult CD4^+^ T cells is the high number of memory CD4^+^ T cells in adults. Therefore, the memory fraction was removed from CD4^+^ T cells and the expression of p21 was measured after exposure of isolated naïve CD4^+^ T cells from newborns and adults (**Figure 4D**) to IFN-β. In accordance with our data derived from total CD4^+^ T cells, IFN-β did not induce p21 in naïve newborn CD4^+^ T cells, whereas IFN-β-mediated induction of p21 was still present in adult naïve CD4^+^ T cells (**Figure 4E**). These data indicate that memory CD4^+^ T cells are not responsible for the induction of p21 in adult CD4^+^ T cells and show an intrinsic difference between naïve CD4^+^ T cells from newborns and adults in which newborns lack the induction of cell cycle inhibitor p21 after exposure to IFN-β.

## Discussion

**Figure 5:**
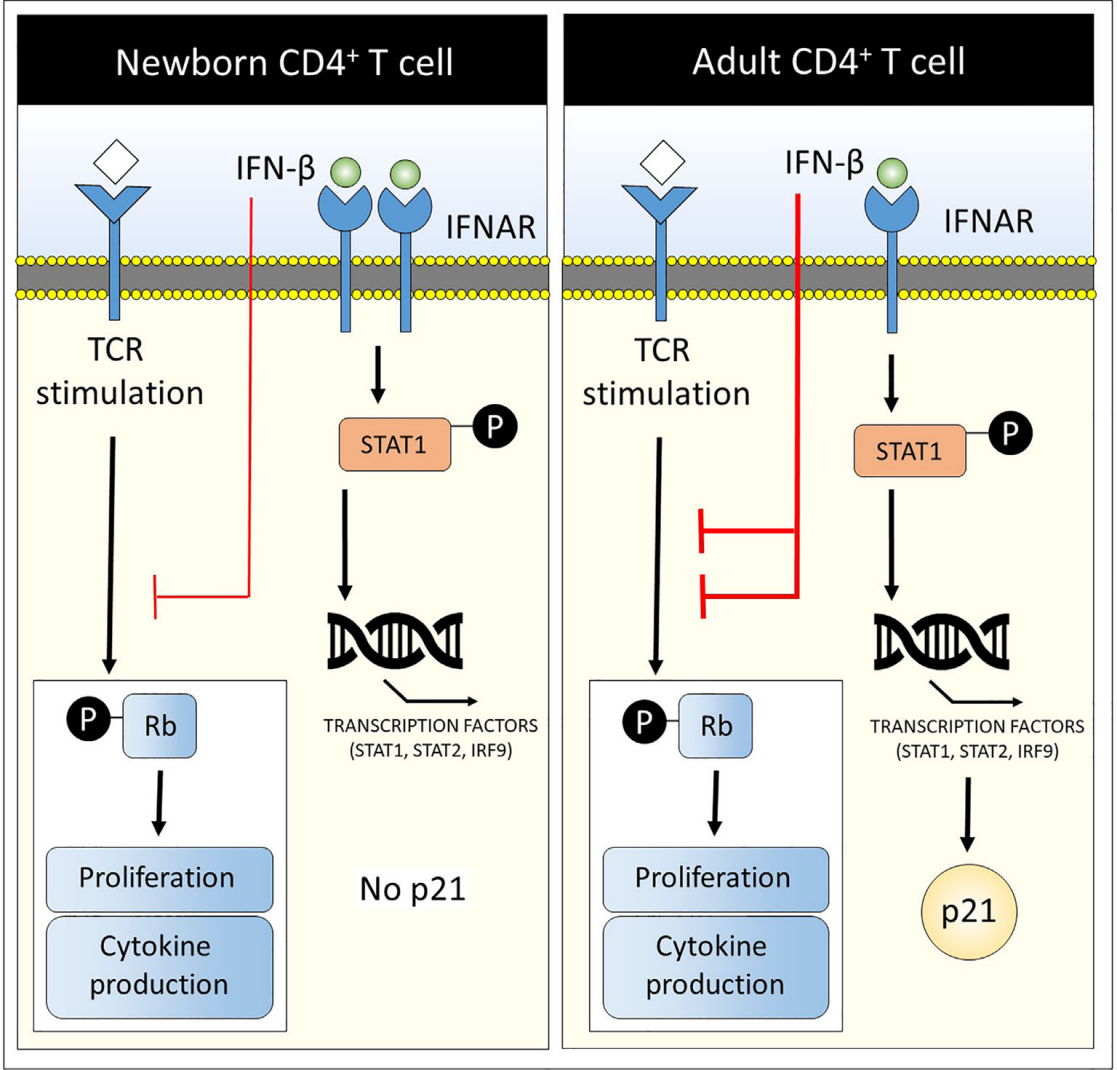
Low CD4^+^ T cell suppression and lack of cell cycle inhibitor p21 in newborns upon exposure to IFN-β. A graphical abstract is depicted that summarizes the main results of this study: Newborn CD4^+^ T cells have a distinct pathway of IFN-β-signaling in which (1) newborn CD4^+^ T cells express higher levels of IFNAR1 and IFNAR2 compared to adults, (2) IFN-β-mediated STAT1 phosphorylation and induction of transcription factors STAT1, STAT2 and IRF9 are comparable between newborn and adult CD4^+^ T cells but (3) newborn total and naïve CD4^+^ T cells, contrary to adults, lack the induction of the cell cycle inhibitor p21 after exposure to IFN-β. In addition, IFN-β has a low inhibitory effect on newborn CD4^+^ T cells with regards to (1) proliferation, (2) phosphorylation of Rb and (3) cytokine production upon TCR stimulation compared to adult CD4^+^ T cells (red lines).

The goal of this study was to determine the differential effects of IFN-β on newborn CD4^+^ T cell functionality compared to adults. It was demonstrated for the first time that IFN-β has a low inhibitory effect on human newborn CD4^+^ T cells with regards to (a) proliferation, (b) phosphorylation of Rb and (c) cytokine production upon TCR stimulation compared to adult CD4^+^ T cells. Newborn CD4^+^ T cells have a distinct pathway of IFN-β-signaling in which (d) newborn CD4^+^ T cells express higher levels of IFNAR1 and IFNAR2 compared to adults, (e) IFN-β-mediated STAT1 phosphorylation and induction of transcription factors STAT1, STAT2 and IRF9 are comparable between newborn and adult CD4^+^ T cells but (f) newborn total and naïve CD4^+^ T cells, contrary to adults, lack the induction of the cell cycle inhibitor p21 after exposure to IFN-β (**Figure 5**).

In this study, we have shown that IFN-β, contrary to IFN-α, inhibits the capacity of newborn and adult T cells to proliferate. Previous reports describe similar differences between IFN-α and IFN-β that may depend on longer receptor binding by IFN-β (17), higher affinity binding by IFN-β (18), differential gene induction by IFN-α and IFN-β (18) or due to the ability of INF-β, and not IFN-α, to induce dimerization of IFNAR1 and IFNAR2 (19). In both our model of MCs and isolated CD4^+^ T cells, IFN-β has a low inhibitory effect on T cell proliferation in newborn CD4^+^ T cells. Therefore, we confirmed our original observation that IFN-β has a low inhibitory effect on newborn T cells compared to adults by looking at multiple effector functions of CD4^+^ T cells, such as cytokine production and phosphorylation of Rb.

The following sequence of IFN-β-mediated signaling was investigated in newborns and adults: Expression of the receptor INFAR1/2, phosphorylation of STAT1 and induction of an important transcriptional complex called ISG factor 3 (ISFG3) that includes the transcription factors STAT1, STAT2 and IRF9 (20). We have shown that these IFN-β-mediated processes are comparable between newborn and adult CD4^+^ T cells. Although the neonatal immune system is considered immature, our data indicate that a substantial part of IFN-β-mediated signaling is comparable between newborns and adults.

A large number of transcriptional factors are transcribed after IFN-β-mediated activation of ISFG3. To elucidate the observed phenotype in which IFN-β exerts an anti-proliferative effect, we specifically examined p21, because it acts as the major effector molecule of cell cycle arrest (14). Previous literature has already shown the causal relationship between the induction of p21 and its anti-proliferative effect on cells (14, 21–23). The requirement of p21 for the anti-proliferative effect of type I IFNs has been observed in cardiomyocytes (21), smooth muscle cells (22), keratinocytes (24) and epithelial cells (25). Although this study did not study the requirement of p21 in the anti-proliferative effect of IFN-β on T cells, we hypothesize that, based on previous literature, the lack of induction of p21 in newborns may explain the low inhibitory effect of IFN-β on newborn CD4^+^ T cells. We first hypothesized that the induction of p21 in adults was a result of the presence of a high number of memory CD4^+^ T cells in adults. However, isolation of naïve CD4^+^ T cells led to similar results in which, again, only newborns lack the induction of p21 after exposure to IFN-β. Many factors contribute to the induction of p21 and can be divided into p53-dependent and p53-independent mechanisms (26). In our model, p53 was not induced on a transcriptional level in adults after exposure to IFN-β, which was comparable with newborn CD4^+^ T cells. However, regulation of p53 also occurs post-transcriptional (27). Therefore, IFN-β may have a differential effect on the post-transcriptional regulation of p53 in newborn CD4^+^ T cells compared to adults. Besides the lack of induction of p21 in newborn CD4^+^ T cells, we also observed a low baseline expression of p21 in naïve newborn CD4^+^ T cells compared to adults which is consistent with the absence of p21 in fetal tissues observed in previous literature (28). From a developmental perspective, low expression of p21 could be essential during a period of growth such as during the fetal and neonatal period. On the other hand, the absent induction of p21 in newborn CD4^+^ T cells could contribute to T cell-mediated immunopathology during infections, because p21 limits overactivation of T cells (29). Differences in expression levels, including the expression of p21, between newborn and adult CD4^+^ T cells could be a result of epigenetic changes, because aging is associated with epigenetic changes. Zhao et al. investigated the epigenome of CD4^+^ T cells from newborns and adults (30). This study did not observe a difference in methylation of p21 between newborn and adult CD4^+^ T cells. Therefore, it is unlikely that epigenetic changes in p21 are responsible for the lack of induction of p21 in newborn CD4^+^ T cells. Interestingly, epigenetic changes between newborn and adult CD4^+^ T cells were observed in the methylation of transcription factors that are involved in the p53-independent regulation of p21. SMAD3, STAT3, STAT5, NEUROD1 are transcription factors that induce p21 in a p53-independent manner and, in addition, are hypermethylated in newborn CD4^+^ T cells compared to adult CD4^+^ T cells (30). Because hypermethylation reduces gene expression, we propose that the lack of induction of p21 in newborn CD4^+^ T cells could be a result of hypermethylation of regions containing SMAD3, STAT3, STAT5 and/or NEUROD1 in newborn CD4^+^ T cells.

It is not yet possible to predict whether the low inhibitory effect of IFN-β on newborn CD4^+^ T cells would be protective or detrimental for disease progression during infection *in vivo*. Proliferation and expansion of CD4^+^ T cells are pivotal for growth and the development of an adaptive immune response after birth and the observed low inhibitory effect of IFN-β could be beneficial for newborns to allow proper growth. On the other hand, neonatal infections can be characterized by exuberant inflammation and newborn CD4^+^ T cells can secrete higher levels of cytokines such as IL-4 compared to adults (31). Low inhibition of newborn CD4^+^ T cells by IFN-β could be responsible for the high production of T-cell-derived cytokines and, thereby, result in a defective control of CD4^+^ T-cell-dependent inflammation leading to inflammation-mediated disease progression *in vivo*.

In conclusion, IFN-β-mediated pathways leading to the induction of STAT1 phosphorylation and the induction of several transcription factors were comparable between newborn and adult CD4^+^ T cells. Signals starting from the induction of p21 such as expression of p21, suppression of Rb phosphorylation and inhibition of the capacity of CD4^+^ T cells to proliferate and produce cytokines were lower in newborns compared to adults. Therefore, the lack of cell cycle inhibitor p21 was pinpointed as an important difference between newborn and adult CD4^+^ T cells during IFN-β-mediated T cell suppression. This study provides novel insights into the regulation of T-cell-mediated immune responses that play an important role during early life infections.

## Data Availability Statement

The raw data supporting the conclusions of this article will be made available by the authors, without undue reservation.

## Ethics Statement

The studies involving human participants were reviewed and approved by Regional Committee on Research involving Human Subjects Arnhem-Nijmegen. Written informed consent for participation was not required for this study in accordance with the national legislation and the institutional requirements.

## Author Contributions

JJ designed the experiments, performed the experiments, including the analyses, and wrote the manuscript. ER, ERS and ME helped perform the experiments including the analyses. WU, RG, MJ and GF helped design the experiments and write the manuscript. All authors contributed to the article and approved the submitted version.

## Conflict of Interest

The authors declare that the research was conducted in the absence of any commercial or financial relationships that could be construed as a potential conflict of interest.

## Supplementary Material

Supplementary Figure 1 IFN-β-mediated inhibition of CD8 ^+^T cell proliferation is reduced in newborns. **(A)** Percentage of proliferating CD8^+^ T cells after incubation of PBMC (adult) or CBMC (newborns) with PHA for 72h. (N=14-19). **(B–E)** CMBCs or PBMCs were pre-incubated with medium, IFN-β **(B, C)**, IFN-α **(D)** or RSV **(E)** for 24h and, subsequently, incubated with PHA for 72h to induce proliferation. Proliferation of CD4^+^ T cells after pre-incubation with medium was normalized for **Supplemental Figures 1C–E**. For this, the proliferation of T cells in the condition with only medium was set to 100%. (N=5-6). (N=5-6). **(F, G)** CBMC **(F)** or PBMC **(G)** were incubated with medium, blocking antibodies against IFNAR2 or isotype controls for 1h followed by exposure to medium or RSV for 24h and PHA for 72h to induce proliferation. Proliferation of CD8^+^ T cells after pre-incubation with medium was set to 100%. (N=5-6). NS = not significant. * = *P*<0.05. ** = *P*<0.01. *** = *P*<0.001.

Supplementary Figure 2 Low bead-induced cytokine production by newborn CD4^+^ T cells and no effect of depletion of Tregs on the inhibitory effect of IFN-β in adults. **(A)** Cell death of newborn and adult CD4^+^ T cells after incubation with medium or IFN-β for 24h followed by incubation with CD3/CD28 beads for 72h. (N=4-6). **(B, C)**. Production of TNF **(B)** and IL-10 **(C)** by newborn and adult CD4^+^ T cells after exposure to CD3/CD28 beads for 72h. (N=5-7). **(D)** Representative samples of CD4^+^ T cells with and without CD25^+^ regulatory T cells (Tregs). **(E)** Adult CD4^+^ T cells and adult CD4^+^ T cells depleted of CD25^+^ cells were pre-incubated with IFN-β for 24h followed by incubation with CD3/CD28 beads to induce proliferation. Proliferation of CD4^+^ T cells after pre-incubation with medium was set to 100%. Data are presented as means ± SEM. * = *P*<0.05. ** = *P*<0.01. *** = *P*<0.001.

Supplementary Figure 3
**(A)** Representative gating to determine IFNAR1 and IFNAR2 expression on CD4^+^ T cell subsets. CD3, CD4 and CD8 was used to identify CD4^+^ T cells and CD8^+^ T cells. CD45RA and CD27 was used to identify T cell subsets: naïve (CD45RA^+^CD27^+^), central memory (CD45RA^-^CD27^+^), effector memory (CD45RA^-^CD27^-^) and terminally differentiated (CD45RA^+^CD27^-^) T cells. **(B)** Percentage of T cell subsets in newborn and adults. (N=4). **(C, D)** Expression of INFAR1 and IFNAR 2 on adult CD4^+^ T cell subsets **(C)** and adult CD8^+^ T cell subsets. (N=5). Data are presented as means ± SEM. * = *P*<0.05. ** = *P*<0.01. *** = *P*<0.001.
