# Supplementary figures and images for: Lack of Cell Cycle Inhibitor p21 and Low CD4+ T Cell Suppression in Newborns After Exposure to IFN-β
Source: Front Immunol. 2021 Apr 12;12:652965. doi: 10.3389/fimmu.2021.652965 (PMC8071872; doi:10.3389/fimmu.2021.652965)

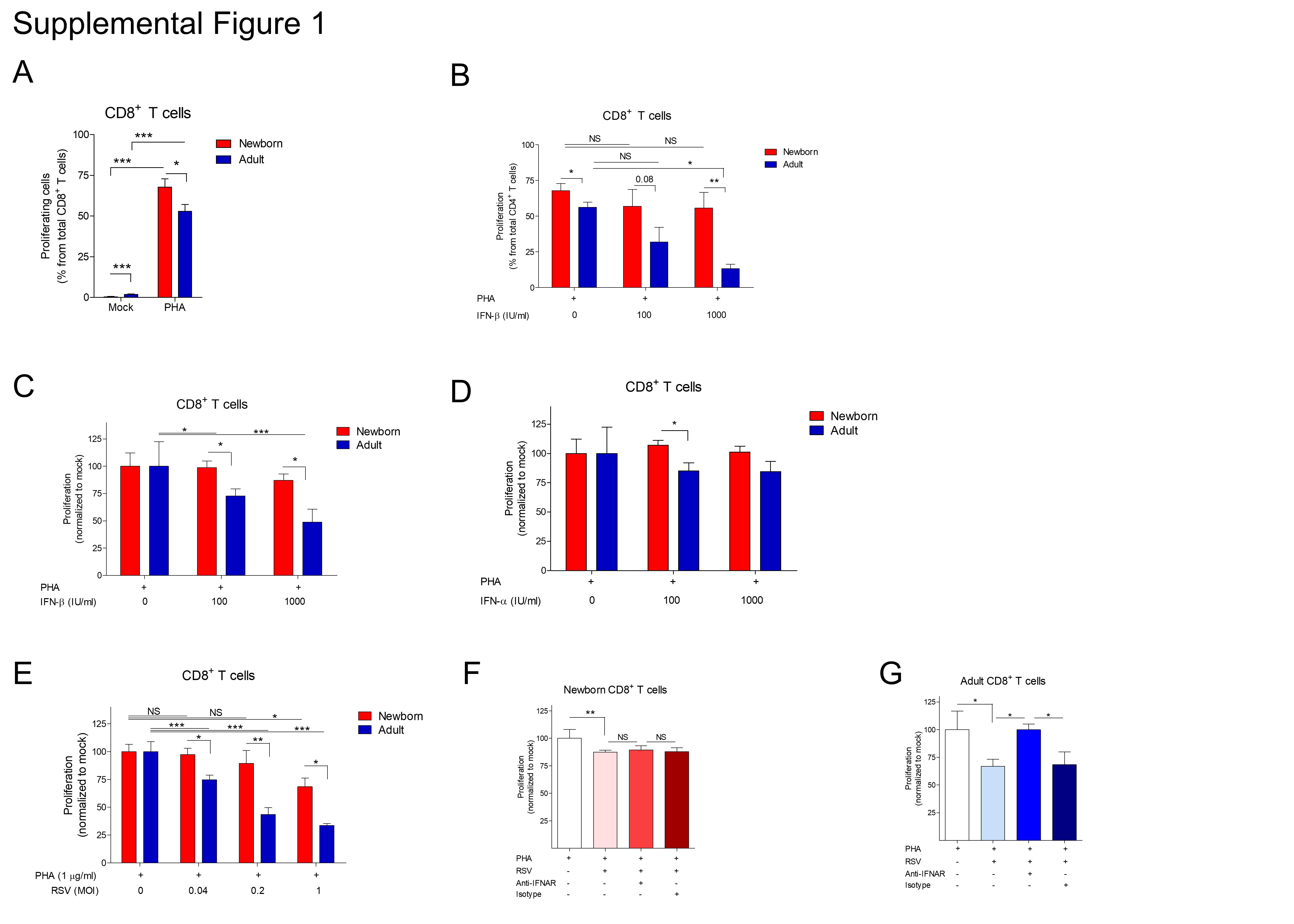

Supplement: Supplementary Figure 1 — IFN-β-mediated inhibition of CD8 +T cell proliferation is reduced in newborns. (A) Percentage of proliferating CD8+ T cells after incubation of PBMC (adult) or CBMC (newborns) with PHA for 72h. (N=14-19). (B–E) CMBCs or PBMCs were pre-incubated with medium, IFN-β (B, C), IFN-α (D) or RSV (E) for 24h and, subsequently, incubated with PHA for 72h to induce proliferation. Proliferation of CD4+ T cells after pre-incubation with medium was normalized for Supplemental Figures 1C–E . For this, the proliferation of T cells in the condition with only medium was set to 100%. (N=5-6). (N=5-6). (F, G) CBMC (F) or PBMC (G) were incubated with medium, blocking antibodies against IFNAR2 or isotype controls for 1h followed by exposure to medium or RSV for 24h and PHA for 72h to induce proliferation. Proliferation of CD8+ T cells after pre-incubation with medium was set to 100%. (N=5-6). NS = not significant. * = P<0.05. ** = P<0.01. *** = P<0.001. [file Image_1.jpg]

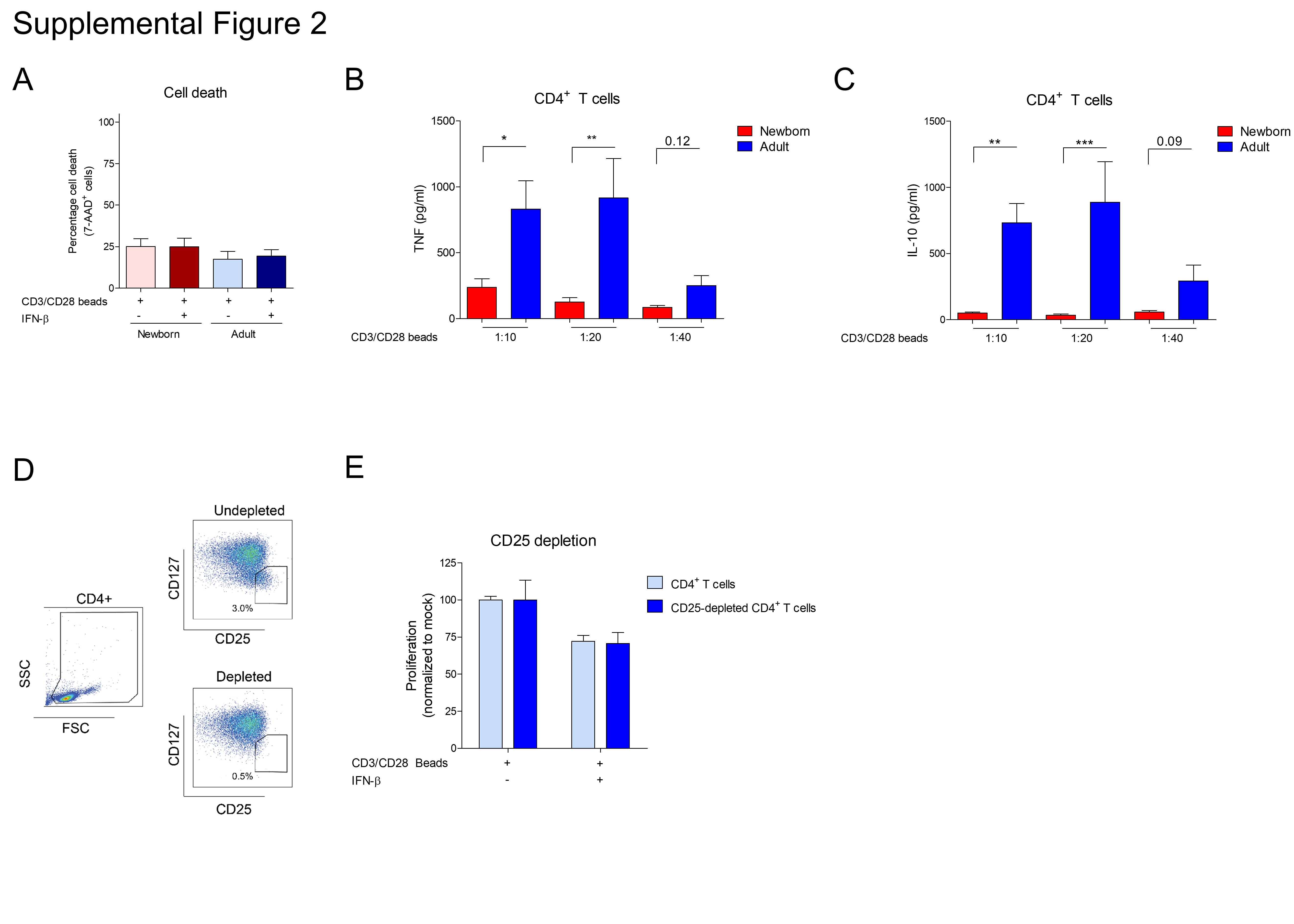

Supplement: Supplementary Figure 2 — Low bead-induced cytokine production by newborn CD4+ T cells and no effect of depletion of Tregs on the inhibitory effect of IFN-β in adults. (A) Cell death of newborn and adult CD4+ T cells after incubation with medium or IFN-β for 24h followed by incubation with CD3/CD28 beads for 72h. (N=4-6). (B, C). Production of TNF (B) and IL-10 (C) by newborn and adult CD4+ T cells after exposure to CD3/CD28 beads for 72h. (N=5-7). (D) Representative samples of CD4+ T cells with and without CD25+ regulatory T cells (Tregs). (E) Adult CD4+ T cells and adult CD4+ T cells depleted of CD25+ cells were pre-incubated with IFN-β for 24h followed by incubation with CD3/CD28 beads to induce proliferation. Proliferation of CD4+ T cells after pre-incubation with medium was set to 100%. Data are presented as means ± SEM. * = P<0.05. ** = P<0.01. *** = P<0.001. [file Image_2.jpg]

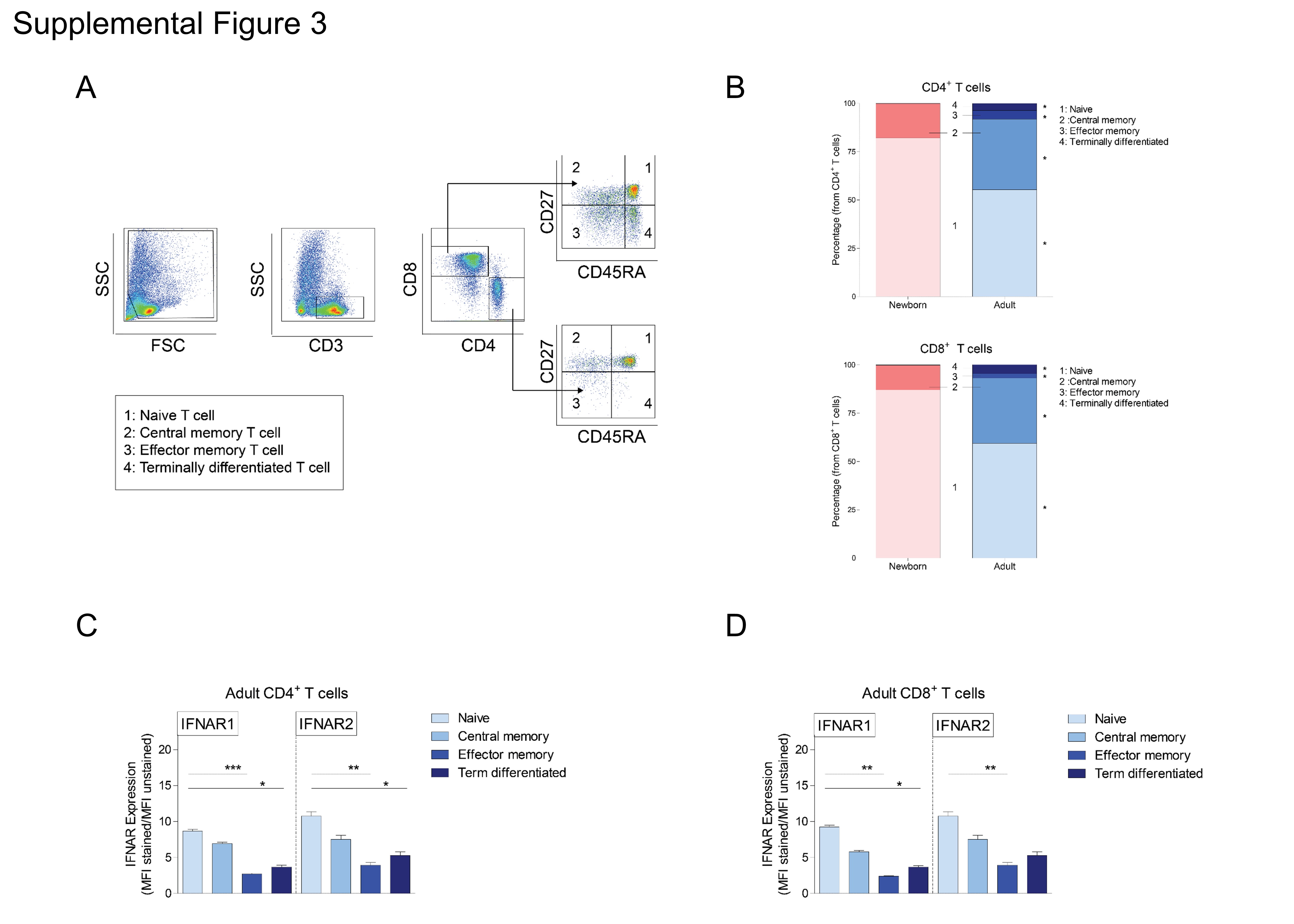

Supplement: Supplementary Figure 3 — (A) Representative gating to determine IFNAR1 and IFNAR2 expression on CD4+ T cell subsets. CD3, CD4 and CD8 was used to identify CD4+ T cells and CD8+ T cells. CD45RA and CD27 was used to identify T cell subsets: naïve (CD45RA+CD27+), central memory (CD45RA-CD27+), effector memory (CD45RA-CD27-) and terminally differentiated (CD45RA+CD27-) T cells. (B) Percentage of T cell subsets in newborn and adults. (N=4). (C, D) Expression of INFAR1 and IFNAR 2 on adult CD4+ T cell subsets (C) and adult CD8+ T cell subsets. (N=5). Data are presented as means ± SEM. * = P<0.05. ** = P<0.01. *** = P<0.001. [file Image_3.jpg]
